# Exploring the potential of *Toxoplasma gondii* in drug development and as a delivery system

**DOI:** 10.1038/s12276-024-01165-7

**Published:** 2024-02-01

**Authors:** Chanjin Yoon, Yu Seong Ham, Woo Jin Gil, Chul-Su Yang

**Affiliations:** 1 Department of Molecular and Life Science, Hanyang University, Ansan, 15588 South Korea; 2 Institute of Natural Science & Technology, Hanyang University, Ansan, 15588 South Korea; 3 Center for Bionano Intelligence Education and Research, Ansan, 15588 South Korea; 4 Department of Medicinal and Life Science, Hanyang University, Ansan, 15588 South Korea

**Keywords:** Parasitic infection, Mechanisms of disease

## Abstract

Immune-mediated inflammatory diseases are various groups of conditions that result in immune system disorders and increased cancer risk. Despite the identification of causative cytokines and pathways, current clinical treatment for immune-mediated inflammatory diseases is limited. In addition, immune-mediated inflammatory disease treatment can increase the risk of cancer. Several previous studies have demonstrated that *Toxoplasma gondii* manipulates the immune response by inhibiting or stimulating cytokines, suggesting the potential for controlling and maintaining a balanced immune system. Additionally, *T. gondii* also has the unique characteristic of being a so-called “Trojan horse” bacterium that can be used as a drug delivery system to treat regions that have been resistant to previous drug delivery therapies. In this study, we reviewed the potential of *T. gondii* in drug development and as a delivery system through current research on inflammation-regulating mechanisms in immune-mediated inflammatory diseases.

## Toxoplasma Gondii: a new hope in treating immune-mediated inflammatory diseases

Immune-mediated inflammatory diseases are a varied set of conditions where the immune system mistakenly attacks the body’s own tissues, causing long-term inflammation. These include illnesses like rheumatoid arthritis, psoriasis, and inflammatory bowel disease. IMIDs have become more common and also raise the risk of certain cancers. They occur due to an imbalance in inflammatory proteins and a misdirected immune response, involving different immune cells and communication pathways, such as the Janus kinase/signal transducer and activator of the transcription pathway. The main findings suggest that *T. gondii* or its antigens could potentially be used to develop new drugs for IMIDs by adjusting the immune response. Additionally, the unique ability of *T. gondii* to cross the blood-brain barrier using immune cells as a “Trojan horse” indicates its potential as a drug delivery tool for hard-to-treat diseases like brain tumors.

This summary was initially drafted using artificial intelligence, then revised and fact-checked by the author.

## Introduction

Immune-mediated inflammatory diseases (IMIDs) are clinically heterogeneous groups of diseases that include rheumatoid arthritis (RA), spondyloarthritis (SpA) disease spectrum, connective tissue disorders, cutaneous inflammatory conditions (including psoriasis and atopic dermatitis), inflammatory bowel disease (IBD), asthma and autoimmune neurological diseases^1^, and their incidence rate has increased over the past several decades^2^. Furthermore, several IMIDs, including IBD, colitis-associated colorectal cancer (CAC), celiac disease, small bowel adenocarcinoma (SBA), primary sclerosing cholangitis, and hepatobiliary cancer, have been associated with an increased risk of developing cancer^3^.

IMIDs are characterized by an imbalance in inflammatory cytokines and dysregulation of the normal immune response. IMIDs share inflammatory pathways initiated by dendritic cells (DCs) and subsets of CD4^+^T cells, such as T helper type 1 (Th1) cells, T helper type 2 (Th2) cells, T helper type 17 (Th17) cells, T follicular helper (Tfh) cells, B regulatory (Breg) cells, and T regulatory (Treg) cells^4–7^. In addition, DCs release interleukin-12 (IL-12) and IL-23 to stimulate Th1 cells, Th17 cells, and Th22 cells to increase the levels of proinflammatory cytokines, including IL-17, interferon-γ (IFN-γ), tumor necrosis factor (TNF) and IL-22^8,9^. Furthermore, a recent study reported that psoriatic patients have elevated levels of inflammatory serum markers, such as beta-defensin 2, lipocalin 2, IL-8, IL-22, and calprotectin^10^. In particular, the expression levels of proinflammatory genes, including CCL2, CCL5, CCL22, VEGF, IL-15, IL-15, TNF, and S100A12, are elevated in the skin and blood of psoriatic patients^9^. Furthermore, recent research has indicated that the Janus kinase/signal transduction and activator of transcription (JAK/STAT) signaling pathway is involved in IMIDs. Diverse proinflammatory cytokines within IMIDs and tumor environments activate JAK/STAT signaling through transduction. The production of type l interferons (IFNs), IL-6, IL-12, and IL-23 is specifically associated with the class I and II cytokine families^11^. For example, Th17 cells, which secrete cytokines such as IL-17 and IL-22, are differentiated by IL-6-activated JAK/STAT signaling^12^. Furthermore, the activation of the JAK/STAT pathway by IL-6 activates different intracellular signaling pathways, including the mitogen-activated protein kinase pathway and the phosphoinositide 3-kinase (PI3K)/protein kinase B (or Akt)/mechanistic target of the rapamycin (mTOR) pathway^13^. In contrast, anti-inflammatory cytokines such as IL-4 and IL-10 can be effective modulators of IMIDs^14,15^.

In this review, we described the immune dysregulation that various IMIDs cause, examined the current treatment and drawbacks of IMIDs, and focused on identifying new drugs available for use in immune-mediated diseases.

### Treatment and drawbacks of IMIDs

Traditional clinical treatments for IMIDs include nonsteroidal anti-inflammatory drugs (NSAIDs), glucocorticoids, and disease-modifying antirheumatic drugs (DMARDs)^16^. NSAIDs are commonly used to treat arthritis and headache and have analgesic, antipyretic, and anti-inflammatory properties^17^. NSAIDs work by inhibiting cyclooxygenase (COX), an enzyme that transforms arachidonic acid into prostaglandins, which are mediators of inflammation and pain^18^. The effects of NSAIDs are analgesic and anti-inflammatory. However, NSAIDs cannot alter the course of IMIDs or prevent damage on their own^19^. Glucocorticoids have potent anti-inflammatory effects and are used to relieve pain and suppress inflammation. However, glucocorticoids are only temporarily used due to their severe side effects^20^. To control the disease process, researchers recommend combining NSAID therapy with DMARDs such as methotrexate (MTX) and leflunomide^21^. MTX is a folic acid-like substance that inhibits intracellular metabolism by preventing the binding of dihydrofolic acid to dihydrofolate reductase^22^. Leflunomide inhibits pyrimidine synthesis and T-cell proliferation^23^, demonstrating an effect comparable to that of MTX in clinical trials. Leflunomide is administered to patients who have contraindications to MTX or who have not demonstrated any MTX-related effects. In addition, combination therapy with leflunomide is highly effective^24^. Several studies indicate that TNF is a therapeutic target in IMIDs^25,26^. Furthermore, anti-TNF medications such as infliximab, adalimumab, etanercept, golimumab, and certolizumab pegol significantly alleviate signs and symptoms in patients with IMIDs^27^. Recently, studies have shown that small molecules that inhibit the intracellular JAK/STAT signaling pathway are bioavailable. Currently, five JAK inhibitors (JAKis), namely, tofacitinib, baricitinib, peficitinib, upadacitinib, and filgotinib, are authorized for use in the treatment of one or more IMIDs^28^. However, all of these treatments have disadvantages and side effects. NSAIDs and selective COX-2 inhibitors increase the risk of stomach ulcers and cardiovascular disease^29^. Glucocorticoids also induce severe adverse effects, including ulcers and bleeding in the gastrointestinal tract, infection, immunosuppression, and bone damage^30^. Furthermore, MTX and leflunomide are associated with hepatotoxicity, pulmonary toxicity, hematologic abnormalities, skin rash, hair loss, weight loss, thrombocytopenia, and diarrhea^31^. Recent research by Li et al. indicated that anti-TNFα biopharmaceuticals increase the risk of serious infections, cardiovascular events, and^32^ cancer. Furthermore, Boyadzhieva et al. confirmed that JAKis have adverse effects, including infectious events, embolism, and thrombosis^33^. Most IMIDs are incurable and are accompanied by complications, including cardiovascular disease, metabolic and bone disorders, and cognitive deficits, which increase mortality^1^. Moreover, IMIDs result from the immune response by aberrant cytokine production, while tumors develop when the immune system does not respond to malignant cells. Therefore, several cancers and cancer treatments have serious adverse effects that increase the risk of IMIDs.

In contemporary medicine, the development of pharmaceuticals faces obstacles such as adverse drug effects, drug toxicity, and drug resistance. Thus, recently, biological drugs derived from the microbiome or parasites have been used to treat various diseases, reduce adverse drug effects and improve treatment outcomes.

### Immune response to *Toxoplasma gondii* infection

**Fig. 1:**
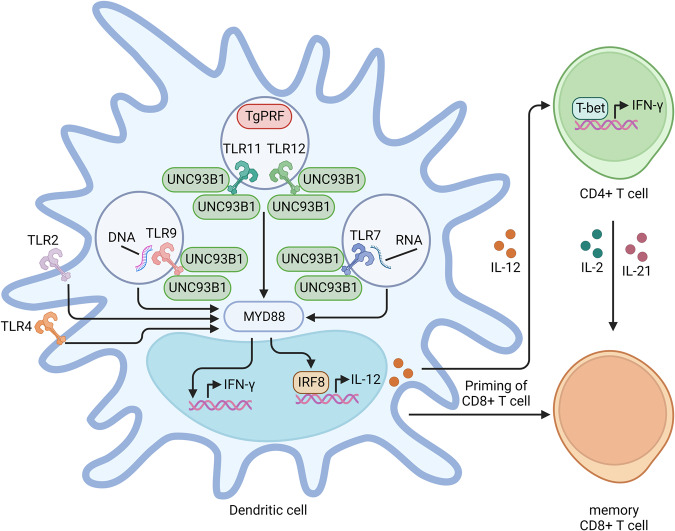
Host immune responses to *T. gondii* infection. TLR11 and TLR12 are the first molecules to recognize TgPRF. TLR7 and TLR9 are receptors for detecting *T. gondii* RNA and genomic DNA, respectively. UNC93B1 binds to endolysosomal TLRs in the endoplasmic reticulum, and stimulation of TLRs activates MyD88-dependent signaling. The MyD88-dependent signaling pathway, which depends on the activation of IRF8, induces IL-12 production in DCs. TLR2, TLR4, and TLR9 play central roles in the production of IFN-γ. However, the production of IL-12 is induced by TLR11 and TLR12. Activated IL-2 induces the differentiation of CD4^+^ T cells into Th1 cells expressing T-bet, which is involved in IFN-γ production. In addition, the production of IL-2 and IL-21 by CD4^+^ T cells helps maintain memory CD8^+^ T cells.

*T. gondii* is a protozoan parasite that infects approximately one-third of the human population worldwide^34^. *T. gondii* causes long-term chronic infection in the tissues of warm-blooded animals and humans and exists as a latent infection state primarily in the tissues of the central nervous system (CNS)^35^. Furthermore, *T. gondii* has developed self-preservation mechanisms to manipulate the immune systems of its hosts, such as by inducing an immune response or immune system evasion. Toll-like receptors (TLRs) are the first to respond to *T. gondii* infection. Gopal et al. reported that the mRNA expression levels of TLR2, TLR4, TLR9, and TLR11 were significantly increased in the MODE-K cell line in response to *T. gondii* *ME49* strain infection^36^. Moreover, Hamie et al. reported that TLR7, TLR11, and TLR12 expression was significantly upregulated in the brains of imiquimod-induced interconversion mice^37^. Another study reported that UNC93 homolog B1 (UNC93B1), which plays an important role in producing IL-12 and IFN-γ, is involved in the function of TLRs (TLR3. TLR7, TLR9, TLR11, TLR12, and TLR13)^38,39^. TLR stimulation activates a signaling cascade involving the TLR-associated adaptor protein MyD88 and the production of IL-12 by dendritic cells (DCs)^40^. The production of IL-12 induces the differentiation of CD4^+^ T cells into Th1 cells expressing the transcription factor T-bet, which is needed for IFN-γ production^41–43^. Interestingly, Benson et al. demonstrated that TLR2, TLR4, and TLR9 play crucial roles in regulating IFN-γ responses in C57BL/6 mice infected with *T. gondii* *ME49*, and TLR11 deficiency has a slight effect on IFN-γ production in *T. gondii* during oral infection. In contrast, the IL-12 response was not elevated by TLR2, TLR4, or TLR9, and only TLR11 activated the production of IL-12 in DCs from *T. gondii* *ME49* strain-infected mice^44^. Moreover, Yarovinsky et al. reported that the activation of the MyD88-dependent downstream immune signaling pathway through TLR11 is associated with TLR12^45^. Raetz et al. demonstrated that TLR11 and TLR12 directly bind to *T. gondii* profilin (TgPRF) and assemble to form a heterodimeric complex and that IFN regulatory factor 8 (IRF8) plays a crucial role in the production of IL-12 through the regulation of TLR11 and TLR12^46^. Koblansky et al. showed that the production of IL-12p40 was decreased in macrophages obtained from TLR12-deficient mice stimulated with TgPRF. Furthermore, the likelihood of *T. gondii* infection was significantly greater in TLR12-deficient mice than in WT mice, and the survival rate was decreased^47^. However, despite TLRs playing a crucial role in the detection of *T. gondii*, TLR11 is a pseudogene, and TLR12 is not expressed in humans^45,48^. TLR7 is a receptor for detecting the RNA of pathogens, such as RNA viruses and bacterial RNA. TLR9 recognizes bacterial and viral DNA containing the cytosine–phosphate–guanine (CpG) dideoxynucleotide motif and induces proinflammatory cytokine production by DCs^45,49^. Numerous studies have reported that the innate and adaptive immune systems respond to *T. gondii* infection by recruiting various cell types, such as inflammatory monocytes, antigen-presenting cells (APCs), T cells, neutrophils (NEs), and natural killer (NK) cells^50,51^. Several in vivo studies have demonstrated the requirement for T cells and the cytokine IFN-γ for preventing parasite reactivation. Disruption of CD8^+^ T cells alone was associated with 50% mortality; in contrast, no mortality was observed when CD4^+^ T cells were exhausted alone^52–54^. Moreover, the maintenance of memory CD8^+^ T cells is dependent on CD4^+^ T-cell help, which is mediated predominantly by IL-2 and IL-21 production^55^. Another study reported that brain pathology or mortality is significantly enhanced in C57BL/6 mice chronically infected with an *ME49* strain of *T. gondii* treated with an anti-CD4 or anti-CD8 mAb alone^52^. In contrast, mice treated with a combination of anti-CD4 and anti-CD8 antibodies showed augmented pathology and mortality similar to those of anti-IFN-γ-treated mice^52^. These results indicate that although CD8^+^ T cells play a dominant role in preventing chronic infection, their maintenance is dependent on the critical help provided by CD4^+^ T cells. CD4^+^ T cells are crucial for the induction of primary CD8^+^ T-cell differentiation by converting APC into an effective stimulator^56^ (Fig. 1). Furthermore, Khan et al. demonstrated that the exhaustion of CD4^+^ T cells by increased expression of the transcription factor BLIMP-1 caused CD8^+^ T dysfunction, leading to the reactivation of latent infection in chronically infected hosts^55^ (Fig. 1). Furthermore, innate immune cells, such as neutrophils, NK cells, and innate lymphoid cells, sequentially and mutually lead to the production of IFN-γ^57^. IFN-γ-mediated immune responses play a crucial role in host protection, and Th2-type immune responses are also involved in resistance to *T. gondii* infection^58,59^. All IL-4-deficient mice died late in toxoplasmosis infection, whereas all control mice survived^59^. A histological study showed that significantly more cysts and areas of acute focal inflammation involved tachyzoites in the brains of IL-4-deficient mice than in those of the control mice in the late stage of *T. gondii* infection^59^. Furthermore, the production of IFN-γ stimulated by *T. gondii* antigens was significantly elevated in the spleen cells of control mice compared with those of IL-4-deficient mice^59^. These results indicate that IL-4 plays a role in inducing IFN-γ production at the late stage of *T. gondii* infection. Additionally, *T. gondii* infection activates nucleotide-binding domain leucine-rich repeat containing receptors (NLRs), which in turn trigger inflammasome assembly and the subsequent activation of caspase-1 and IL-1β secretion, resulting in pyroptosis in various cell types^60^.

Interestingly, to resist the immune response, *T. gondii* has successfully evolved strategies to evade the host immune system and migrate to immune-privileged sites, such as the CNS. *T. gondii* prevents the host immune system from entering through multiple mechanisms, including transcriptional regulation and interference with cell signaling, resulting in the secretion of inflammatory cytokines, modulation of matrix metalloproteases (MMPs), production of microbicidal molecules and apoptosis^61–64^. In particular, *T. gondii* secretes effector proteins, including rhoptry proteins (ROPs) and dense granules (GRAs), from secretory organelles; these proteins alter host cell signaling pathways and protect *T. gondii* throughout infection^65^.

### *T. gondii*-associated diseases

Primary and chronic *T. gondii* infection is generally harmless and asymptomatic in most immunocompetent individuals^66^. However, *T. gondii* infection is most dangerous during pregnancy and in people with compromised immune systems, such as those with human immunodeficiency virus (HIV)^67^. If a pregnant woman contracts a primary infection, it is transmitted to the fetus through the amniotic fluid and placenta, resulting in congenital toxoplasmosis^68^. Congenital toxoplasmosis is responsible for fetal death and infant hydrocephalus, cerebral calcifications, and ocular toxoplasmosis (OT)^69^. Typically, prenatally acquired infections frequently cause OT. However, several studies have reported that postnatally acquired infection is also associated with OT^70,71^. The clinical presentation of OT depends on the anatomical location of the lesion^72^. Furthermore, de-la-Torre et al. reported that specific cytokine patterns, such as inflammation, recurrence, and infection with a *T. gondii* strain, are correlated with the clinical presentation of OT^73^. Knight et al. reported that IL-4, IL-6, CCL2, CXCL-2, and CXCL-8 were increased in Müller cells infected with *T. gondii RH* or the *Prugniaud* strain. In contrast, IFN-γ and IL-12 expression is not altered by *T. gondii* infection^74^. Furthermore, various studies have reported that the levels of Th1 cells and inflammatory cytokines, such as IL-2, IL-6, IL-17, and IFN-γ, are elevated in the aqueous humor of OT patients. In contrast, the Th2 cytokine IL-13 was poorly expressed^73^.

Acute infection and reactivation of *T. gondii* are associated with multiple sclerosis (MS) and neurological disorders by inducing inflammatory lesions in the CNS in patients with severely compromised immune systems. MS is an inflammatory disease of the CNS that is primarily driven by a chronic autoimmune response. In recent years, several studies have reported an association between *T. gondii* infection and MS incidence. Except for one study, four out of five studies revealed a negative association between *T. gondii* infection and MS; however, one study that reported a positive association had an insufficient sample size, particularly because of a lack of significance. More recently, Nicoletti et al.^75^ demonstrated a significant negative association between *T. gondii* seropositivity and MS in a population-based case‒control study that included 164 patients and 481 controls. Furthermore, Cicero et al. reported a significant negative association between *T. gondii* and MS via meta-analysis. The incidence of clinical symptoms and the expression levels of cytokines such as TNF-α and INF-γ were lower in *T. gondii RH* strain-infected MS mice than in MS mice^76^.

Parkinson’s disease (PD) and Alzheimer’s disease (AD) are the most common age-related neurodegenerative disorders. Numerous epidemiological and experimental studies have demonstrated the association between *T. gondii* infection and the development of PD and AD. Associations between *T. gondii* and PD were based on data collected from eight studies. Two of the eight studies showed a significant association based on *T. gondii* infection determined via IgG-ELISA, and one revealed a significant association based on *T. gondii* infection defined through PCR. However, an increase in the incidence of *T. gondii* infection in PD patients compared with that in control individuals might not indicate a risk factor for PD. In fact, PD patients are at increased risk of *T. gondii* infection due to decreased personal and food hygiene habits. Furthermore, Çelik et al. and Oskouei et al. did not observe significant differences between *T. gondii* infection and PD^77,78^. Several studies have suggested that *T. gondii* infection is a risk factor for the development of AD. Galeh et al. investigated the effects of chronic *T. gondii* infection on cognitive impairment in an amyloid beta 1–42 (Aβ1-42)-induced AD rat model established with the Types I (*RH*), II (*PRU*), and III (*VEG*) strains and in combination. The RH strain plays a detrimental role in AD pathogenesis. In contrast, chronic infection with the *PRU* strain or a combination of the *PRU* and *VEG* strains significantly improved cognitive deficits in Alzheimer’s disease rat models^79^.

In summary, the possible association between *T. gondii* infection and disease risk has still not been fully characterized, and conversely, a negative correlation between *T. gondii* infection and disease status has been reported. Therefore, these results should be interpreted cautiously, and further studies are needed to confirm the association between *T. gondii* and disease.

### Anti-inflammatory effects of *T. gondii*

Multiple studies have indicated that *T. gondii* modulates the cytokine response by either inhibiting or augmenting it, resulting in an antagonized immune response. Recently, Charles et al. reported that CD4^+^ T-cell activation is suppressed by the cell surface protein programmed death ligand 1 (PD-L1) in mouse retinal pigment epithelial cells and leukocytes^80^. Additionally, Washino et al. reported that IL-17 mRNA expression is inhibited in CD4^+^ T cells isolated from the mesenteric lymph nodes and spleen of IL-1Ra-deficient induced-RA model mice infected with *T. gondii*. Moreover, the mRNA expression of RORγt, a key transcription factor for Th17 cells on CD4^+^ cells, was decreased in infected mice compared to control mice, indicating that *T. gondii* regulates transcription factors to reduce Th17 cell polarization^81^. Furthermore, Kim et al. reported that the ratio of CD4^+^ cells, macrophages, and NK cells classified as splenocytes is significantly reduced in *T. gondii*-infected mice^82^. Similarly, Butcher et al. demonstrated that *T. gondii*-infected macrophages do not produce TNF-α or IL-12 compared to lipopolysaccharide (LPS)-treated peritoneal macrophages and several macrophage lines, including J774, RAW264.7, and THP-1 cells. Furthermore, LPS-induced TNF-α production was reduced in macrophages infected with *T. gondii*^83^. In addition to its effect on macrophages, LPS stimulation does not fully increase TNF-α levels in *T. gondii*-infected NEs compared to those in *T. gondii*-infected NEs treated with LPS alone^84^.

**Fig. 2:**
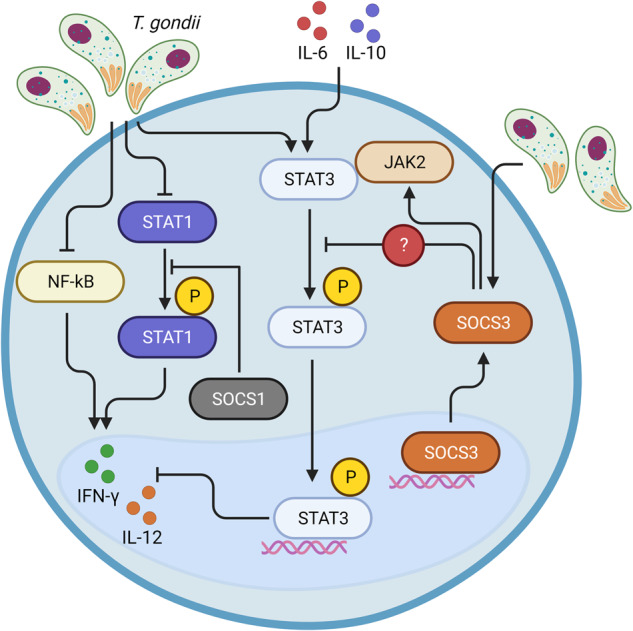
Mechanisms of antagonizing the host immune response by *T. gondii*. After invasion of the host cell, *T gondii* alters host signaling pathways and gene expression, antagonizing immune responses. STAT3 activated by IL-6 and IL-10 is associated with antagonizing host defense. *T. gondii* also directly activates STAT3 to antagonize the host immune response. *T. gondii* also upregulates SOCS3, which binds JAK-2 and inhibits the phosphorylation of STAT3. The depletion of SOCS3 enhanced *T. gondii*-induced STAT3 signaling, resulting in reduced levels of IL-12 and IFN-γ. Taken together, these results indicate that SOCS3 might have additional mechanisms to limit the phosphorylation of STAT3. In addition, *T. gondii* antagonizes IFN-γ production by interfering with NF-κB activation and STAT1 activation, which are inhibited by SOCS1.

Several studies have indicated that *T. gondii* stimulates the secretion of anti-inflammatory cytokines. Yan et al. reported an increase in IL-4 mRNA expression and secretion in *T. gondii*-infected RAW264.7 macrophages compared to those in the control group^85^. Hunter et al. demonstrated that the mRNA expression of IL-4 was detected concurrently with the onset of inflammation 10 days after infection with the *RRA* strain of *T*. gondii in the brains of C57BL/10 Sc Sn mice. Furthermore, of all the mice, 2 were still IL-4 positive on Day 20^86^. Suzuki et al. reported that *T. gondii* cysts, foci of acute inflammation, and tachyzoite-specific mRNAs were significantly more common in the brains of IL-4^−^^/^^−^ mice than in those of controls in the early stage of *T. gondii* infection^59^. In the early stages of *T. gondii* infection, the complex reaction of rapidly dividing tachyzoites and the immune response can often lead to host death, and IL-4 plays a protective role by reducing the products of Th1 cells^87^. Another study reported that IL-4, IL-10, and transforming growth factor-β (TGF-β) levels gradually and significantly increased in the *T. gondii* acute infection stage of C57BL/6 mice. Furthermore, the transcript levels of anti-inflammatory cytokines (IL-4, IL-10, and TGF-β) were increased in *T. gondii*-infected mouse brains compared with those in control mouse brains^88^. Additionally, compared with that in noninfected control mice, IL-10 expression in the intestines of infected mice was elevated^89^. Furthermore, Bliss et al.^90^ demonstrated that the expression of IL-10 was detected by confocal fluorescence microscopy in *T. gondii*-infected macrophages and confirmed that IL-10^+^ cells were F4/80^+^ by FACS. Elevated levels of IL-10 were detected in the lymph nodes and CNS of CBA/Ca mice that developed progressive toxoplasmic encephalitis (TE)^91^. Beaman et al. reported that STAT3 is activated by IL-6 and IL-10, which antagonize the defense mechanism and result in enhanced susceptibility to toxoplasmosis. Furthermore, activated STAT3 reduces IL-12 production to evade immunosurveillance; however, *T. gondii* also directly activates STAT3 to escape host immunity. *T. gondii* also upregulates SOCS3, which blocks the phosphorylation of STAT3 by inhibiting JAK-2 directly. The depletion of SOCS3 resulted in elevated *T. gondii*-induced STAT3 signaling, and macrophages and neutrophils with SOCS3 deletion had reduced levels of IL-12 and IFN-γ and succumbed to toxoplasmosis. These results indicate that SOCS3 might have additional mechanisms to restrict the phosphorylation of STAT3. In addition, *T. gondii* antagonizes IFN-γ production by interfering with NF-κB activation and STAT1 activation, which are inhibited by SOCS1^92^ (Fig. 2). Zhang et al. demonstrated that IL-5 expression is increased in the brain and spleen of C57BL/6 mice infected with the ME49 strain of *T. gondii* compared with those in the control group. Moreover, IL-5 KO mice exhibited decreased survival rates and weight loss during *T. gondii* infection^93^. In contrast, Nickdel et al. reported that, compared with WT mice infected by the same route, IL-5-deficient mice infected with the *RRA* strain *T. gondii* through the oral route had significantly greater survival rates, irrespective of sex. Moreover, splenocytes obtained from WT mice produced significantly more IL-12 and IFN-γ than those obtained from IL-5^−/−^ mice when stimulated with TLA. Conversely, compared with those in WT mice, the plasma IL-12 and IFN-γ levels in IL-5-deficient mice infected with *T. gondii* orally were significantly greater on Days 6 and 8, respectively^94^. These results indicate that IL-5 plays different roles in influencing the immune response and disease progression between acute and chronic *T. gondii* infection. TGF-β is an anti-inflammatory cytokine that plays an important role in antagonizing TNFα, TNFβ, IFNγ, and IL-12^95,96^. Several studies have reported that TGF-β-deficient mice develop inflammatory autoimmune disease^97–99^. Nagineni et al. reported that TGF-β1 and TGF-β2 mRNA levels are increased in *T. gondii*-infected human retinal pigment epithelial cultures (HRPE). Furthermore, soluble extracts of *T. gondii* significantly increase the secretion of both TGF-β1 and TGF-β2^100^.

In summary, *T. gondii* infection leads to anti-inflammatory cytokine production through an altered host immune system by manipulating host gene transcription and regulating signaling pathways.

### Reversing the immune response by *T. gondii*

In recent decades, multiple studies have indicated that inflammation is involved in tumorigenesis. Chronic inflammation initiates tumor progression through genetic modifications, such as the accumulation of DNA mutations, epigenetic alterations, or both^101^. Furthermore, inflammatory cytokines, such as TNF-α, IL-1, and IL-6; growth factors; chemokines; and proteases secreted by tumor-associated lymphocytes and macrophages increase tumor cell proliferation, survival, migration, and invasion, thus promoting tumor growth and metastasis^102^. Furthermore, tumor-associated macrophages (TAMs) produce inflammatory mediators involved in tumor angiogenesis and lymphangiogenesis^103^ and secrete immune-suppressive soluble cytokines, including TGF-β and IL-10^104^. Interestingly, inflammation has a questionable benefit in relation to cancer. In contrast, TNF functions as an endogenous tumor promoter. TNF inhibits tumor growth by inducing apoptosis and inhibiting nuclear factor κB (NF-κB) signaling^105^. Furthermore, cell death through CD8^+^ cytotoxic T cells and natural killer (NK) cells, as well as tumor cell senescence induced by CD4^+^ Th1 cells that produce IFN-γ and TNF-α, inhibits tumor growth^106^. Another study revealed that IFN-γ signaling and antigen presentation are essential for the lysis mediated by CD8^+^ cytotoxic T cells in mouse colon carcinoma cells and that TNF is a key factor in antitumor activity^107^. Consequently, the immune response reawakened by *T. gondii* can increase susceptibility to cancer treatment or lead to the development of an effective treatment.

According to previous research, immune cells, including NEs, DCs, macrophages, and T cells, produce TNF-α in response to *T. gondii* infection. Bliss et al. reported that NEs treated with the *T. gondii* antigen produced IL-12p70 and TNF-α. Additionally, elevated TNF-α induces macrophage-inflammatory MIP-1α and MIP-1β gene expression via autocrine stimulation in *T. gondii*-infected human peripheral blood neutrophils^108^. Additionally, *T. gondii* recruits NEs through a multitude of immature DC chemotactic chemokines. Furthermore, *T. gondii*-cascade neutrophils release chemokines that induce DC CD40 and CD86 upregulation in a manner dependent on *T. gondii*-induced neutrophil TNF-α production^109^. The ovarian tumor protease (OTU) domain, ubiquitin aldehyde binding 1 (OTUB1), and deubiquitinase increase in DCs after infection with *T. gondii* and LPS stimulation. Mulas et al. demonstrated that TgPRF and LPS-induced activation of NF-κB increased the production of IL-6, IL-12, and TNF-α in DCs^110^. Similarly, Corrêa et al. reported that *T. gondii* infection increases the expression of proinflammatory cytokines, including IL-12, IL-1β, IFN-γ, and TNF-α, in macrophages from P2X7^+^^/^^+^ mice^111^. Mennechet et al. demonstrated that IFN-γ and TNF-α levels were elevated in primed lamina propria CD4^+^ T cells isolated from mice infected with *T. gondii*. Furthermore, the expression of C-C chemokine mRNAs, such as RANTES, monocyte chemoattractant protein-1 (MCP-1), and MCP-3, and CXC chemokine mRNAs, such as IP-10, was significantly upregulated in response to infection with *T. gondii*^112^.

Although the suppressive effect of the increased immune response induced by *T. gondii* infection in different cancer types has not yet been fully elucidated, several studies have shown that *T. gondii* contributes to host antitumor immunity. Wu et al. demonstrated that *T. gondii* infection induces apoptosis and inhibits cell proliferation in various cancer cell lines, including the breast cancer cell line MCF-7, prostate cancer cell line DU145, esophageal cancer cell line EC-109, and lung cancer cell line A549^113,114^. In addition, a previous study showed that *T. gondii* infection modulates the expression of tumor-related factors, thus improving the antitumor capacity of hosts. TP53 is a tumor suppressor gene that is frequently mutated in various cancers. *T. gondii* infection upregulates the expression of Ladd45, which induces cell cycle arrest and apoptosis and is involved in the p53 signaling pathway. This study showed that DCC, Smad3, Smad4, hMLH1, hMSH2, and hMSH3, which are involved in the colorectal cancer pathway, were regulated by *T. gondii* infection. Furthermore, *T. gondii* infection increases the RASSF1 tumor suppressor gene in non-small cell lung cancer (NSCLC) patients. After *T. gondii* infection, the expression of the breast cancer-related genes CCND1 and PRCA2 decreased and increased, respectively^115^.

**Fig. 3:**
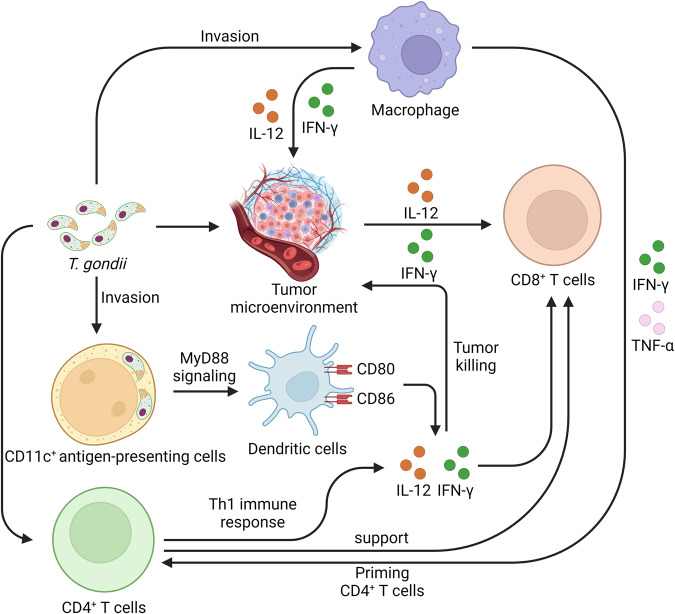
Mechanisms of reversing tumor-associated immunosuppression with *T. gondii* vaccination *T*. *gondii* infection might reverse the immunosuppressive state of the TME to stimulate effective therapeutic immunity. *T. gondii* recruits macrophages that produce proinflammatory cytokines, and the produced IFN-γ and TNF-α enhance CD4^+^ T-cell priming. In addition, *T. gondii* invades CD11c^+^ APCs and converts DCs to a CD80- and CD86-expressing immunostimulatory phenotype via MyD88 signaling. IL-12 and IFN-γ production restricts tumor growth through enhancement of CD8^+^ T-cell infiltration. In addition, *T. gondii* infection significantly enhances infiltrating CD4^+^ T-cell activation and helps maintain CD8^+^ T-cell activation. Finally, macrophages, CD4^+^ T cells, and CD8^+^ T cells are recruited to the TME to restrict tumor growth synergistically via secreted IL-12 and IFN-γ.

Several studies have reported the application of nonreplicating *T. gondii* uracil auxotrophs (NRTUAs), which are considered “immunotherapy attenuated vaccine strains”, in cancer treatment. Although NRTUAs are nontoxic and are rapidly cleared by the immune system in approximately 5 days, they are still able to significantly inhibit tumor progression even after they are killed. Baird et al. reported that NRTUAs with carbamoyl phosphate synthetase II (CPS-II) knockout invade CD8^+^ T cells and NK cells to induce antitumor immune activity. NRTUA treatment altered IL-12, IFN-γ, and the CXCR3-stimulating cytokine-involved CD8^+^ T-cell population in the tumor microenvironment (TME), subsequently inhibiting melanoma tumor growth in mice^116^. Moreover, *RH*-Δ*cps* predominantly invades immunosuppressive CD11c^+^ APCs and converts to a CD80- and CD86-expressing immunostimulatory phenotype in the ovarian carcinoma microenvironment. In response to increased levels of T-cell receptor costimulatory molecules, CD11c^+^ cells reawaken the ability to cross-present and prime CD8^+^ T cells. Moreover, *RH*-Δ*cps* treatment significantly reduced the tumor size and enhanced the survival rate of ovarian tumor-bearing mice. These results demonstrated that *T. gondii* effectively inhibits ovarian tumor progression by restoring the T-cell population in tumor-bearing mice^117^. Sanders et al. reported that *RH*-Δ*cps* treatment significantly enhances infiltrating CD4^+^ and CD8^+^ T-cell activation and elevates the number of circulating tumor-specific T cells in the TME in pancreatic cancer patients. Furthermore, *RH*-Δ*cps* treatment had significant therapeutic effects on pancreatic tumor-bearing mice, as indicated by changes in IL-12 and IFN-γ production, MyD88 signaling, and CD8^+^ T-cell populations^118^. Another NRTUA, orotidine-5’-monophosphate decarboxylase (OMPDC) knockout, revealed vaccine features that might induce significant immune responses by enhancing the expression of inflammatory cytokines such as IL-12p40 and IFN-γ in mouse serum and the TME. Moreover, *RH*-Δompdc restricted tumor growth, reduced lung metastasis, and increased the survival rate of 4T1 breast tumor-bearing mice^119^. Bahwal et al. reported that the combination of *RH*-ΔompdcΔup and an anti-PD-1 antibody induced a significant antitumor immune response and synergistically restricted tumor growth through the enhancement of CD8^+^ T-cell infiltration induced by DC-secreted IL-12 and IFN-γ production in pancreatic ductal adenocarcinoma (PDAC) tumors^120^. Li et al. reported that *ME49*-Δompdc inhibits the growth of B16F1 melanoma by eliciting a Th1 immune response, with high levels of IL-12, TNF-α, and IFN-γ and CD8^+^ T-cell activation in C57BL/6 mice^121^ (Fig. 3).

In conclusion, *T. gondii* has multiple functions associated with host and antitumor immune responses by reversing tumor-associated immunosuppression. However, because the pathological mechanisms differ among various carcinomas, further research is needed to fully understand the antitumor effect of *T. gondii*.

### Immune response to *T. gondii* antigens

*T. gondii* microneme proteins (MICs), ROPs, GRAs, surface antigens (SAGs), and SAG-1-related sequences (SRSs) are antigens that play crucial roles in parasite infection and maintaining intracellular host-parasite relationships. First, the MICs located at the tip of *T. gondii* mediate the motility and adhesion of the host cell. When the parasite is transmitted to a host cell, the apical membrane antigen 1 (AMA1)-rhoptry neck protein (RON) pairs form a moving junction, and the subsequent ROPs involved in the formation of the parasitophorous vacuole membrane (PVM) are secreted simultaneously with invasion. After the invasion and establishment of the PVM, GRA secretion continues and is involved in the maturation of the PVM and in the maintenance of host-parasite interactions for intracellular survival and replication^122–124^.

Recent studies have reported that *T. gondii* antigens influence the immune response. Benevides et al. showed that IFN-γ and TNF-α levels were significantly elevated in the ileum of *T. gondii*-infected mice [76]. However, the levels of the IFN-γ and TNF mRNAs were lower for the soluble antigen of *T. gondii* tachyzoites (STAg) than for those of *T. gondii*-infected mice. Furthermore, pretreatment with soluble STAg decreased the number of CD4^+^ T cells expressing proinflammatory cytokines in the lamina propria (LP) and increased the number of CD8^+^ T cells in the intraepithelium of the small intestine^125^. Moreover, STAg increases the expression of suppressor of cytokine signaling 2 (SOCS2) in DCs through the action of endogenous lipoxin A4 (LXA4), which is dependent on 5-lipoxygenase (5-LOX), resulting in a reduction in the secretion of IL-2 and in the secretion of C-C chemokine receptor 5 (CCR5)^126^. According to another study, TNF-α release and IL-1β secretion are reduced by pretreatment of LPS-stimulated Ana-1 macrophages with *T. gondii* excretory/secretory antigens (TgESAs)^127^. These findings suggest that ThAgs, a component of *T. gondii*, modulate immune modulation. MIC1 and MIC4 interact with TLR2 and TLR4 and activate NF-κB to induce the expression of proinflammatory cytokines, such as IL-12, TNF-α, and IL-6, in mouse bone marrow-derived macrophages (BMDMs). Interestingly, MIC1 and MIC4 also stimulate the secretion of the anti-inflammatory cytokine IL-10 by transporting TLR4 into endosomes, although the underlying mechanisms are unknown^128^.

Recently, Chen et al. demonstrated that ROP16 interacts with STAT3 and STAT6 in host cells to promote their phosphorylation, resulting in a cascade of downstream signals for host cell manipulation^129^. The phosphorylation of STAT6 by ROP16 induces arginase-1 (Arg-1), which is involved in anti-inflammatory effects, tumor immunity, and immunosuppression-related diseases^130^. Arg-1 is also a key effector and marker of M2a macrophages through STAT3 and STAT6^131^. M2 macrophages secrete anti-inflammatory cytokines, such as IL-10 and IL-1ra, which inhibit the host immune response to *T. gondii* infection^81,89,132^. Furthermore, ROP16 promotes the differentiation of M2 macrophages through STAT6 and STAT3 activation by inducing IL-4 and IL-10, respectively^133^. Specifically, ROP16 types I and III (ROP16 I/III) activate M2 macrophages to ameliorate IBD^134^. Similarly, the deletion of ROP5, ROP18, and GRA7 was associated with resistance to IFN-γ-activated immunity-related GTPases (IRGs), such as Irgm1/m3, which degrade PV to promote parasite clearance, resulting in increased PV clearance and presentation of soluble PV antigen by MHC I molecules in macrophages or DCs from C57BL/6 mice^135^. Kim et al. reported that GRA9 has the potential to ameliorate sepsis by increasing anti-inflammatory and antibacterial effects through the conversion of M1 macrophages into M2 macrophages. Furthermore, GRA9 inhibits NLRP3 inflammasome assembly and suppresses the activation of pro-IL-1β and pro-IL-18 in BMDMs^136^. The *T. gondii* antigens SAG-1 and SAG-2 inhibit the mRNA expression of Th1 cytokines and proinflammatory cytokines, including IL-1, IL-6, GM-CSF, and TNF-α. Furthermore, the levels of SAG-1 and SAG-2. increased. Treatment with *T. gondii* infection and an anti-IFN-gamma mAb inhibited the expression of TNF-alpha and iNOS^137^.

**Fig. 4:**
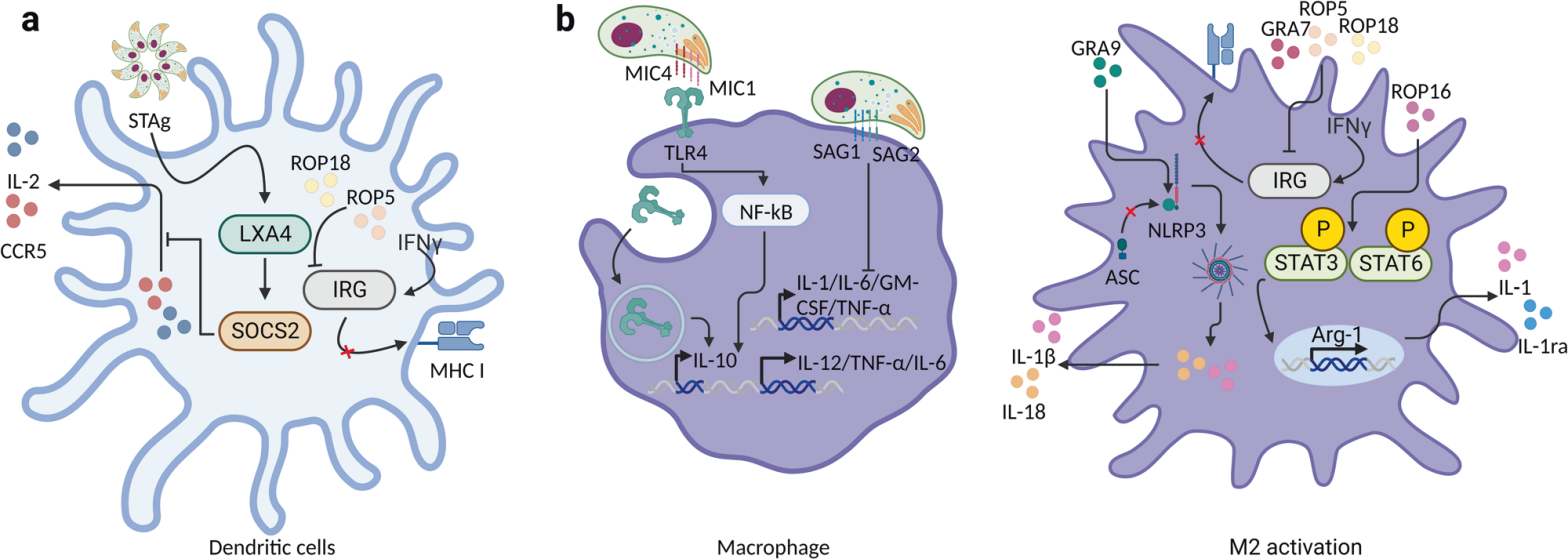
The *T. gondii* antigen alters the signaling pathway of the host cell. *T. gondii* antigens, including MICs, ROPs, GRAs, and SAGs, manipulate host cell signaling pathways. **a** In DCs, STAg induces the expression of SOCS2 via LXA4 and consequently suppresses IL-2 expression and CCR5 secretion. ROP5 and ROP18 suppress MHC I antigen presentation by resisting IFN-γ and IRG. **b** In macrophages, MIC1 and MIC4 bind to TLR4; induce the secretion of IL-12, TNF-α, and IL-6 through NF-κB; and translocate TLR4 to endosomes to produce the anti-inflammatory cytokine IL-10. SAG-1 and SAG-2 induce IL-1/IL-6/GM-CSF/TNF-α transcription in macrophages. GRA9 disrupts NLRP3 inflammasome assembly by blocking the binding of ASC to NLRP3 and consequently suppressing IL-1β and IL-18 secretion. ROP5, ROP18, and GRA7 inhibit MHC I antigen presentation by resisting IFN-γ and IRG. ROP16 activates STAT3- and STAT6-dependent M2 macrophages and induces the expression of the M2 macrophage marker Arg-1 and the secretion of IL-1 and IL-1ra.

Several studies have indicated that *T. gondii* antigens stimulate an antitumor immune response. PVM-associated *T. gondii* antigens, such as ROP5, ROP17, and ROP18, are essential for inducing an immune response. Furthermore, although the antitumor mechanisms of ROP35 and ROP38 are independent of the ROP5, ROP17, and ROP18 complexes, PVM-associated ROP35 and ROP38 are essential for the antitumor response. Furthermore, GRA2, GRA12, and GRA24 significantly inhibited the antitumor response to ovarian tumors^138^. Li et al. reported that GRA15 induced M1 macrophage differentiation; suppressed multiple properties of hepatocellular carcinoma, including proliferation, migration, and invasion; and decreased the expression of MMP-2 and MMP-9. In addition to tumor tissues and growth suppression, TNF-α and IL-12 mRNA levels are increased in splenocytes, while the expression of the IL-6 and IL-10 mRNAs is downregulated^139^. Another study reported that GRA15 decreases cell viability and induces apoptosis in JEG-3 choriocarcinoma cells by regulating genes associated with apoptosis and endoplasmic reticulum stress genes^140^. Seo et al. reported that GRA16 inhibits upstream Akt signaling and suppresses NF-B signaling in a PP2A-dependent manner. In NSCLC, cotreatment with irinotecan, an anticancer drug, and GRA16 induced cell cycle arrest and inhibited cell proliferation^141^ (Fig. 4).

In summary, antigens are expected to play an important role in regulating the immune response of *T. gondii*. However, despite the presence of numerous antigens of *T. gondii*, only immune responses to a few antigens have been identified. Therefore, further research on the function of other *T. gondii* antigens and their underlying mechanisms is needed.

### A unique characteristic of *T. gondii* for drug delivery

Drug development is a costly and time-consuming process. In an effort to improve the safety and efficacy of previously developed drugs, numerous studies, including studies on drug delivery systems (DDSs), have been conducted. DDSs aim to efficiently deliver drugs to target locations, minimize adverse effects, and improve safety and efficacy to achieve the maximum therapeutic effect. Drug delivery consists of two concepts: first, a prodrug that has no medicinal effect outside the body but is metabolized by metabolic enzymes when it enters the body; and second, the direct delivery of drugs to target molecules based on a DDS^142^. Similarly, the second concept consists of a DDS using a polymer such as a synthetic polymer, micelle, liposome, or antibody and a DDS using convergence technology. Although the development of convergence technology has affected DDSs, novel technologies are still needed.

However, further studies are needed to develop new DDSs for the treatment of dementia, Parkinson’s disease (PD), and brain tumors, which are all diseases of the CNS caused by abnormalities or degeneration. These diseases are often difficult to treat via drug delivery. The blood‒brain barrier (BBB), a crucial immunological feature of the human CNS, plays a crucial role in blocking external toxic substances and restricting drug permeation^143^. In addition to brain diseases, the TME also has difficulties in drug delivery. The reason is that tumor blood vessels that supply nutrients to tumors are more prevalent at the tumor and host border than in central regions^144^. Therefore, the injection of drugs through blood vessels has a major limitation; drugs are delivered only to the periphery of blood vessels and rarely reach the center of the tumor.

**Fig. 5:**
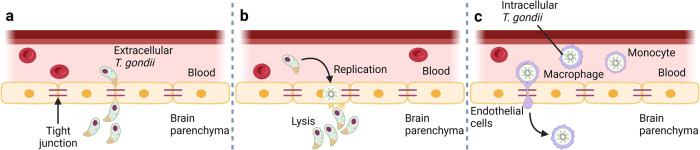
Mechanisms for the transfer of *T. gondii* from the blood to the brain. Three modes by which *T. gondii* invades the CNS from the blood. **a** *T. gondii* accesses the brain by disrupting tight junction proteins. **b** Direct infection and lysis of endothelial cells in the BBB by *T. gondii*. **c** The “Trojan horse” strategy, trafficking within infected immune cells.

A unique characteristic of *T. gondii* is its ability to metastasize to various organs, including the eyes, heart, liver, lungs, lymph nodes, and muscles. Furthermore, *T. gondii* can reach the brain, invade the CNS, and cause TE, establishing a continuous chronic infection in nerves and other brain cells^145,146^. There are three possible ways that *T. gondii* invades the CNS from the blood. First, in the BBB paracellular pathway, *T. gondii* tachyzoites accumulate in the peripheral circulation and cell-free *T. gondii* tachyzoites cross the BBB by disrupting tight junction proteins^145,146^. Second, during BBB endothelial cell lysis, free *T. gondii* and *T. gondii* released from positive monocytes can invade cerebrovascular endothelial cells (ECs), replicate in ECs, and subsequently impair the entry of ECs into the CNS^147,148^. The third way is to hijack immune cells; during viral or bacterial infections or neuroinflammatory diseases, DCs enter the perivascular, cerebrospinal fluid (CSF), and parenchymal regions of the brain through the BBB^149^. *T. gondii* successfully uses DCs and monocytes as vehicles for protection across the BBB and invasion of the brain^150,151^. Courret et al. demonstrated that spread from the intestine to the brain is predominantly mediated by CD11c^−^ CD11b^+^ monocytes, which are preferentially infected via the blood and, to a lesser extent, CD11c^+^ CD11b^+^^/^^−^ DCs^152^. This migration within infected immune cells is called the “Trojan horse” strategy (Fig. 5).

Recent research has focused on immune cells as a drug delivery mechanism. Immune cells migrate within the TME and interact with other immune cells^153^. Circulating cell-mediated drug delivery, often specifically termed the Trojan horse strategy, has led to new strategies for delivering drugs to tumor centers or delivering drugs to difficult regions using host immune cells. For example, peripheral blood leukocytes are preferentially recruited to the TME, where they enhance tumor progression by inducing tissue remodeling and affecting angiogenesis^154^. NEs, the most abundant type of leukocyte, are reported to infiltrate inflamed brain tumors. Xue et al. demonstrated that liposomes that contain paclitaxel (PTX) and use NEs as “Trojan horses” can cross the BBB and suppress the relapse of glioma in mice whose tumor has been resected surgically^155^. Furthermore, He et al. reported that inflammatory monocyte-based biomimetic drug delivery systems loaded with legumain-activated nanoparticles inhibit the proliferation, migration, and invasion of breast cancer cells and penetrate metastatic tumors, resulting in significantly inhibited delivery to lung metastases^156^. Choi et al. demonstrated that nanoparticle-containing monocytes differentiated into macrophages and that the nanoparticle-laden macrophages penetrated the center of the T47D tumor spheroid. This result indicated that the tumor environment recruits monocytes and that nanoparticle-based drug delivery through monocytes might be exploited for therapeutic purposes^157^. Moreover, Lee et al. reported the use of inflammatory CD11b^+^ cells as active carriers to deliver doxorubicin-loaded nanoparticles into poorly vascularized regions of tumors^158^.

Although the application of Trojan horse drugs is promising, current therapy depends on adoptive cell transfer. In adoptive cell therapy, target cells are extracted from internal organs, cultivated ex vivo, and subsequently infused into patients^159^. However, ex vivo isolation and expansion consume time, and according to Marrache et al.^160^, the cytokine response of monocytes or macrophages is not always reproducible, preventing practical clinical translation. Furthermore, innate immune cells, such as NEs and monocytes, generally exhibit short lifespans that are less than expected in vivo^161^. To resolve these issues, drugs need flexible, functional groups that can be injected directly into the blood circulation and, depending on the target, conjugated to specific or diverse cell carriers.

Thus, the ability of *T. gondii* to infect various immune cell types, such as monocytes, DCs, and macrophages, and to infect Trojan horses has demonstrated its potential as a drug delivery tool.

## Conclusion

IMIDs are a broad group of diseases caused by immune imbalances, such as abnormal cytokine production. However, currently, no definitive clinical treatment for IMIDs exists. Additionally, IMID treatment, which suppresses abnormal cytokine elevation, may increase the risk of developing cancer. The evidence provided in this manuscript leads to the conclusion that, due to their immunomodulatory capacity, *T. gondii* or *T. gondii* antigens could be new materials for treating IMIDs. First, *T. gondii* acts like a double-edged sword. *T. gondii* infection enhances the production of anti-inflammatory cytokines. However, conversely, NRTUA vaccination effectively controls tumor growth by reversing tumor-associated immunosuppression through an increase in proinflammatory cytokines. Furthermore, *T. gondii* antigens, such as MIC, ROP, and GRA, play roles in both stimulating the production of proinflammatory cytokines and inhibiting proinflammatory cytokines. Taken together, these findings suggest that *T. gondii* may maintain a balanced immune system by inducing a more stable and controlled immune response. Second, *T. gondii* has unique characteristics for drug delivery. *T. gondii* can infiltrate the BBB using immune cells, as in Trojan horses. Drug delivery via *T. gondii* employs immune cells that are involved in various diseases, and this approach might be applied to various diseases for which existing drug delivery has been difficult, including cancer. However, despite the presence of numerous *T. gondii* antigens, few *T. gondii* antigens have been identified. Thus, further studies are needed on the immune response to additional *T. gondii* antigens or synergistic effects of antigens and their molecular mechanisms. Ultimately, understanding and applying the immune response regulatory mechanisms of *T. gondii* may provide new perspectives on the potential for developing drugs for inflammatory diseases, cancer, and other intractable diseases.
